# A health equity monitoring framework based on process mining

**DOI:** 10.1371/journal.pdig.0000575

**Published:** 2024-08-28

**Authors:** Jan Niklas Adams, Jennifer Ziegler, Matthew McDermott, Molly J. Douglas, René Eber, Judy Wawira Gichoya, Deirdre Goode, Swami Sankaranarayanan, Ziyue Chen, Wil M. P. van der Aalst, Leo Anthony Celi

**Affiliations:** 1 Chair of Process and Data Science, RWTH Aachen University, Aachen, Germany; 2 Laboratory for Computational Physiology, Massachusetts Institute of Technology, Cambridge, Massachusetts, United States of America; 3 Department of Internal Medicine, Section of Critical Care, University of Manitoba, Winnipeg, Manitoba, Canada; 4 Department of Biomedical Informatics, Harvard Medical School, Boston, Massachusetts, United States of America; 5 Department of Surgery, University of Arizona, Tucson, Arizona, United States of America; 6 Montpellier Research in Management, Montpellier University, Montpellier, France; 7 Department of Radiology & Imaging Sciences, Emory University, Atlanta, Georgia, United States of America; 8 Computer Science and Artificial Intelligence Laboratory, Massachusetts Institute of Technology, Cambridge, Massachusetts, United States of America; 9 Genome Institute of Singapore (GIS), Agency for Science, Technology and Research (A*STAR), Singapore; 10 Fraunhofer Institute for Applied Information Technology, Sankt Augustin, Germany; 11 Division of Pulmonary, Critical Care and Sleep Medicine, Beth Israel Deaconess Medical Center, Boston, Massachusetts, United States of America; 12 Department of Biostatistics, Harvard T.H. Chan School of Public Health, Boston, Massachusetts, United States of America; Mayo Clinic Rochester: Mayo Clinic Minnesota, UNITED STATES OF AMERICA

## Abstract

In the United States, there is a proposal to link hospital Medicare payments with health equity measures, signaling a need to precisely measure equity in healthcare delivery. Despite significant research demonstrating disparities in health care outcomes and access, there is a noticeable gap in tools available to assess health equity across various health conditions and treatments. The available tools often focus on a single area of patient care, such as medication delivery, but fail to examine the entire health care process. The objective of this study is to propose a process mining framework to provide a comprehensive view of health equity. Using event logs which track all actions during patient care, this method allows us to look at disparities in single and multiple treatment steps, but also in the broader strategy of treatment delivery. We have applied this framework to the management of patients with sepsis in the Intensive Care Unit (ICU), focusing on sex and English language proficiency. We found no significant differences between treatments of male and female patients. However, for patients who don’t speak English, there was a notable delay in starting their treatment, even though their illness was just as severe and subsequent treatments were similar. This framework subsumes existing individual approaches to measure health inequities and offers a comprehensive approach to pinpoint and delve into healthcare disparities, providing a valuable tool for research and policy-making aiming at more equitable healthcare.

## Author summary

The US is pushing major legislative efforts to make health equity a primary factor in the reimbursement of health institutions. This raises the question of how to measure health equity. Specifically, how to measure health equity within these care-delivering healthcare institutions that are not affected by existing institutional inequities outside the care-delivering institution. We introduce a process mining framework that assesses equity in the care-delivering processes. This means we investigate how different treatments are conducted, how they are timed, and how the general care strategy is allocated. This framework equips regulatory bodies with a toolkit to assess the equity of delivered care within an institution.

## Introduction

Legislation in the United States (US) has recently proposed tying Medicare reimbursement to the health equity of the treatments provided [1] and including health equity standards into the hospital accreditation program of the joint commission [2]. This legislation is motivated by the well-established health equity research and care disparities that have been reported previously, such as racial differences in prenatal care [3] or worsened outcomes based on linguistic disparities [4]. These outcome disparities are subject to regular reporting [5] and causes have been broken down into the factors of epigenetic racial differences, cultural, preference, implicit provider biases, and structural inequalities [6].

Linking reimbursement to health equity requires a means to measure the health equity of delivered care. While individual studies exist to measure health equity for care delivery of specific diagnoses and treatments, there is a lack of tools available to measure inequities in delivering healthcare. Furthermore, it is unclear what causes health inequity. Studies on health equity offer mixed insights. While some find no notable treatment disparities after accounting for confounders [7–11], others detect differences in treatment without adverse outcomes for minorities [12]. However, a few link treatment disparities directly to negative outcomes such as delayed ICU transfers [13] and lesser preventive care [14], or lesser subspecialty care [15].

One major limitation of the current literature is that only single steps of a care pathway are investigated, such as ventilation or antibiotics treatment. This demonstrates the central limitation that while confounders outside of the care process are accounted for, e.g., socioeconomic status, age, sex, comorbidities, illness severity, etc., the confounders within the care process are often left out, e.g., previous and subsequent treatment steps, their length, and time delay [8]. Using more granular clinical data may, therefore, allow for adjustment of these important factors in assessing inequities in care provision.

Process mining is a computer science discipline analyzing the data of end-to-end process executions [16]. It builds on event logs, which collect all executed actions during the instantiation of a process, including the predecessor and successor steps of a single treatment. Currently, process mining has been used to optimize operational processes within hospitals [17, 18] and to learn models of patient treatment pathways of patients [19–21]. Process mining has not been utilized to investigate biases and inequities in delivering care. Using the event log, single-treatment step inequities, inequities arising for multiple, dependent treatment steps and inequities on the global level of treatment allocation can be assessed using process mining. In this paper, we introduce a process mining approach to construct a general framework for measuring health inequities. This method not only highlights single-treatment step inequities but also captures disparities in multiple interdependent treatment steps and the broader treatment allocation strategy and can be used as a general pipeline to investigate inequities in care delivery. Sepsis management is a treatment process that is characterized by its complexity and dependency on timely responses. Therefore, we apply this framework to assess the inequities in care for patients with sepsis in the ICU attributed to sex and English language proficiency and demonstrate the applicability of our framework.

## Method

**Fig 1:**
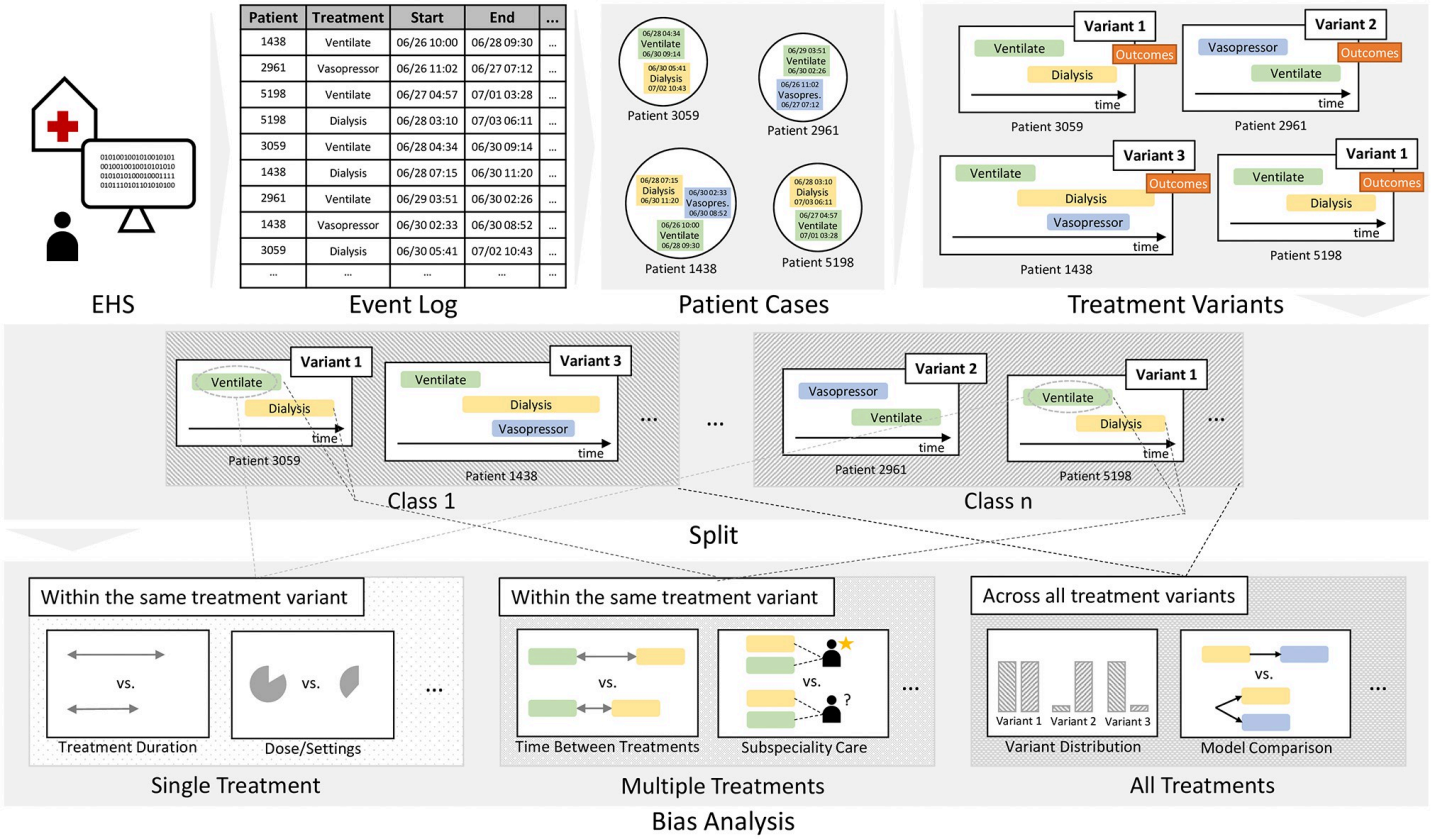
Framework overview. Overview of the framework to assess bias in treatments. The treatment variants along with the outcomes of all patients are extracted from the event log of the EHS. Subsequently, the variants are split according to a sensitive attribute, and we conduct bias analysis for single treatments, multiple treatments, and the global allocation of treatments.

The proposed framework is an end-to-end solution that can be applied to an Electronic Health System (EHS) and provides various health inequity measures as an output. There are two major steps in the framework. First, the data is extracted into an event log that contains treatment information, and each treatment is referred to as a treatment step. Next, we split the event log patient based on different markers of health inequity and investigate for disparities with respect to single treatment steps, multiple treatment steps, or the global distribution of treatments. The workflow of the framework is shown in Fig 1.

### Data preparation

The data is prepared by homogenizing patients by diagnosis and comorbidities, then identifying treatments, and creating an event log.

#### Homogenizing patients

First, the primary patient diagnosis is identified and patients not conforming to this diagnosis are filtered out. We conduct a two-step procedure to control for comorbidities that can be applied based on the availability and size of the data set: First, we identify specific comorbidities correlated with high negative outcomes, and, second, we split patient groups according to similar comorbidities with high negative outcomes.

#### Identifying treatments

Treatment identification involves linking entries in the database of the EHS to the occurrence of a treatment and its associated data. This step is conducted by domain experts who can identify relevant treatments and their attributes for the corresponding diagnosis. Additional attributes are extracted. The conducted treatments are associated with patients through the database information.

#### Creating the event log

The resulting data are extracted into a table where each row describes an event, containing a patient identifier and the administered treatment, including start and end time, the amount administered, and machine parameters. Additionally, the event log contains a patient data table with the personal attributes for each patient. We group all events that are associated with the same patient into a so-called case. The case provides the full set of treatment steps associated with a single patient. We extract the outcomes for each patient and associated them with the specific case. In the following, we introduce variants which are the abstraction of a case to its order of treatments.

#### Treatment variants

The specific order of starting and ending different treatment steps is called a *treatment variant*. The treatment steps of each patient are ordered by time of occurrence to retrieve the treatment variant of each case. Two cases belong to the same treatment variant if the order in which their treatments started and ended is the same. Subsequently, the data is prepared for bias analysis by splitting the variants into multiple groups according to a chosen marker of health inequity, e.g., ethnicity, language, age, or sex.

### Bias analysis

The core part of our framework is bias and disparity analysis. We subdivide this into three different types of biases that can be analyzed: single-treatment step biases, multiple-treatment steps biases, and global treatment biases. Each of these three categories contains several specific biases. Our framework is extensible to new or custom disparity analysis that local data experts wish to measure.

#### Single-treatment step biases

Single-treatment step biases focus on the manifestation of a single part of the overall treatment for all patients that follow the same treatment variant. The aggregated values of a characteristic of the considered single treatment step are compared. This can be any characteristic of the treatment, such as the amount of administered antibiotics. In our baseline version of the framework, we include the average time for the considered treatment step. Furthermore, we compute the average SOFA score at the start of the treatment as a control variable.

#### Multiple-treatment steps biases

Multiple-treatment step biases are differences between patient groups in the relationship between multiple treatment steps within the same treatment variant. The aggregated statistics for a collection of treatment steps are calculated. An example would be the time between to treatment steps, e.g., the time between admission and first administration of antibiotics. In our baseline version of the framework, we calculate the average time between subsequent treatments.

#### Global treatment biases

Global treatment biases are differences between patient groups in the global allocation of treatment sequences. Techniques that can provide a comparison between distributions of patients over variants are applied to the variants. If the variant distribution is not the same, there are differences in either the administration of certain treatments or their ordering between patient groups, e.g., lower likelihood of ventilation. In our baseline framework, we use Pearson’s chi-square test [22] with the null hypothesis of variant distributions being equal between two patient groups. We conduct a pairwise comparison between patient groups.

#### Uncertainty estimation

There needs to be an uncertainty estimation of the generated statistical measurements. In our baseline version of the framework, these are generated by incorporating bootstrapping. Through sampling and recalculating statistics, we provide confidence intervals that provide a sense of the reliability of the individual measure. The comparison of distributions is supported through hypothesis testing, providing significance levels for the difference in distributions between patient groups. In the baseline version of the framework, determine the 90% confidence intervals of all metrics by bootstrapping through sampling. These metrics include times, attributes, and outcomes. We depict confidence intervals using the mean and the allowed deviations to stay within the 90% confidence interval.

## Results

### Cohort development

The Medical Information Mart for Intensive Care IV (MIMIC-IV) [23], a single-center database containing de-identified structured and free-text electronic health record (EHR) data from patients admitted to Beth Israel Deaconess Medical Center, 2008–2019 was used. A cohort of all adult (≥18 years age) patients admitted to an ICU with a primary diagnosis of sepsis and with one of the three comorbidities: congestive heart failure, myocardial infarct, or chronic pulmonary disease, were selected. Demographics including age, sex, self-reported race, language proficiency (English-speaking or non-English speaking), comorbidities, as well as treatment events (see below), the start time of each treatment event, the patient’s Sequential Organ Failure Assessment (SOFA) score at the start time of the event, and the end time of the event were extracted for each patient. All code is publicly available on GitHub (https://github.com/niklasadams/CareDisparities). This study is reported in accordance with the STrengthening the Reporting of OBservational studies in Epidemiology (STROBE) statement (cf. S1 Fig).

MIMIC-IV has been previously approved for research use by the Beth Israel Deaconess Medical Center Institutional Review Board. All individuals involved in this study completed the required data and specimens human subjects research training and signed the requisite data use agreement.

For the event log, we extract treatment events that correspond to mechanical ventilation, the administration of vasopressors, and renal replacement therapy. The SOFA scores at each event are the 24-hour SOFA scores at the start of the event. If there is no available SOFA value, we use the next available.

We determine the treatment variant for each patient and filter out infrequent variants of less than 50 patients as these lack statistical support for further analysis. We received 24 variants. We depict the top 5 variants with aggregated attributes for sex and language in S4 Fig through S13 Fig. On the final variant set, perform two splits according to the sensitive attributes of sex and language.

### Bias analysis

#### Patient sex

The descriptive statistics for the age, English proficiency, and ethnicity of female and male patients are depicted in Table 1. There was no significant difference in the distribution of treatment variants by sex (cf. S2 Fig). With a p-value of 0.035, we cannot reject the null hypothesis of equal distributions at a significance level of 1% and 2.5%.

**Table 1:** Baseline characteristics of patient sex.

	Female (n = 4481)	Male (n = 6151)	p-value
Age, median (IQR)	70 (19)	67 (19)	<0.05
English speaking, n (%)	4043 (90%)	5573 (91%)	0.53
Race/ethnicity			
White	2955 (66%)	4279 (70%)	<0.05
Black	592 (13%)	457 (7%)	
Hispanic/Latino	143 (3%)	193 (3%)	
Asian	103 (2%)	165 (3%)	
Native American	10 (0.2%)	9 (0.1%)	
Other	678 (15%)	1048 (17%)	

**Fig 2:**
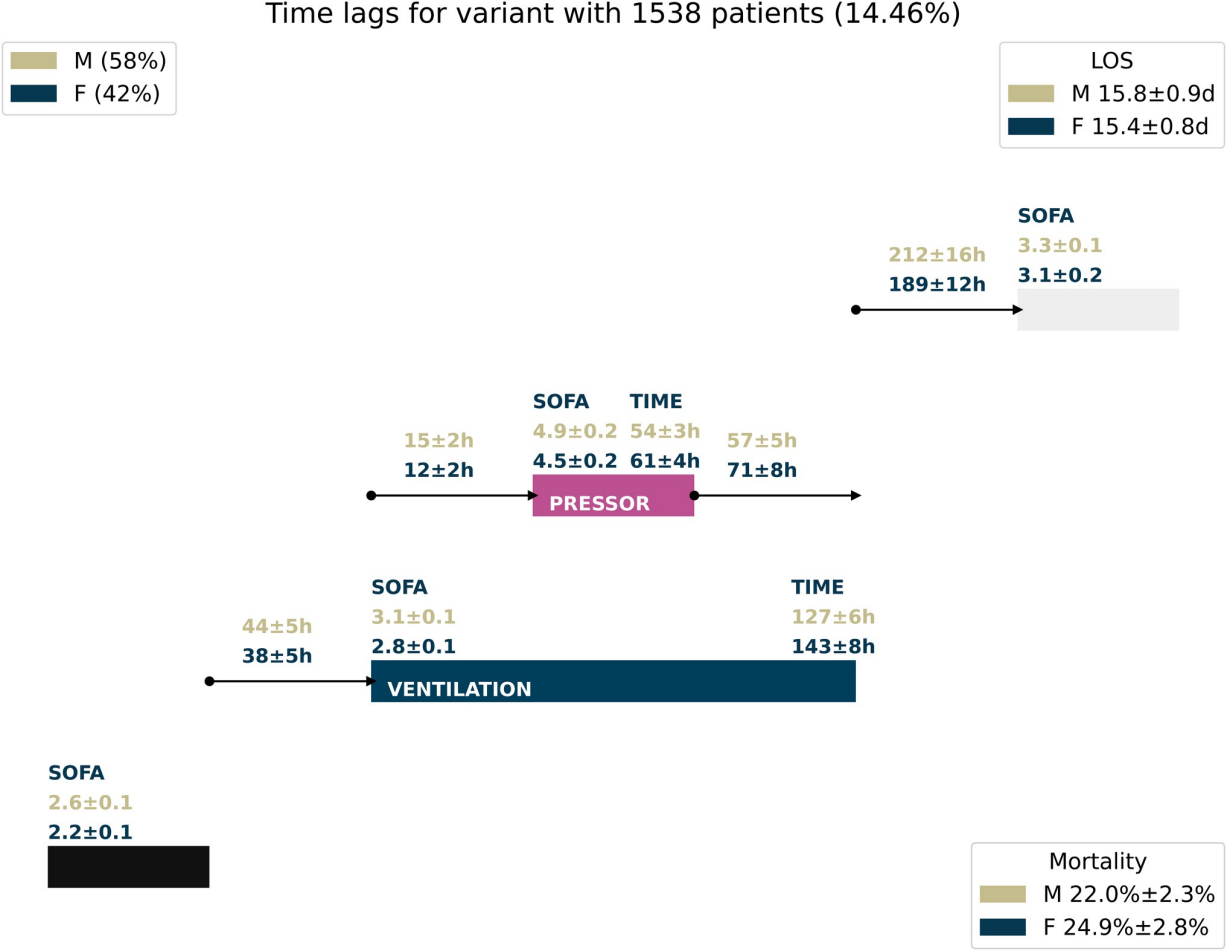
Treatment variants with metrics for male/female patients. Average treatment durations, time differences, and SOFA scores for one treatment variant, compared for male/female patients. 90% confidence intervals are depicted.

For multiple- and single-treatment step analysis, we investigate the third most frequent treatment variant depicted in Fig 2 with large statistical support of 1538 patients. This treatment variant describes the following treatment sequence: After ICU admission, the patients are ventilated. After the start of ventilation, the patients receive vasopressor. The administration of vasopressor is stopped at some point in time, afterward, the ventilation is also stopped. At last, the patient is discharged. The SOFA score of both male and female patients is in a similar range upon admission, which also holds for the time until the ventilation is started. For the remaining treatments, we observe similar times of treatments and times between treatments. There are only minor trends of female treatment times being longer while the times between treatments are shorter. This holds true even though the average SOFA score of female patients is slightly below that of male patients. The outcomes also do not contain large disparities between male and female patients. Overall, we cannot observe any significant sex-based disparity in this treatment variant.

#### Language

The descriptive statistics for the age, sex, and ethnicity of English and non-English speaking patients are depicted in Table 2. There was a significant difference in the distribution of treatment variants by sex (cf. S3 Fig). With a p-value of 0.00025, we can reject the null hypothesis at any typical significance level.

**Table 2:** Baseline characteristics of English-speaking (ESP) and non-English-speaking (non-ESP) patients.

	ESP (n = 9616)	Non-ESP (n = 1016)	p-value
Age, median (IQR)	68 (19)	73 (20)	≪ 0.05
Male sex, n (%)	5573 (58%)	578 (57%)	0.53
Race/ethnicity			
White	6899 (72%)	335 (32%)	≪ 0.05
Black	966 (10%)	210 (21%)	
Hispanic/Latino	126 (1%)	83 (8%)	
Asian	104 (1%)	164 (16%)	
Native American	19 (0.2%)	0 (0%)	
Other	1502 (16%)	224 (22%)	

**Fig 3:**
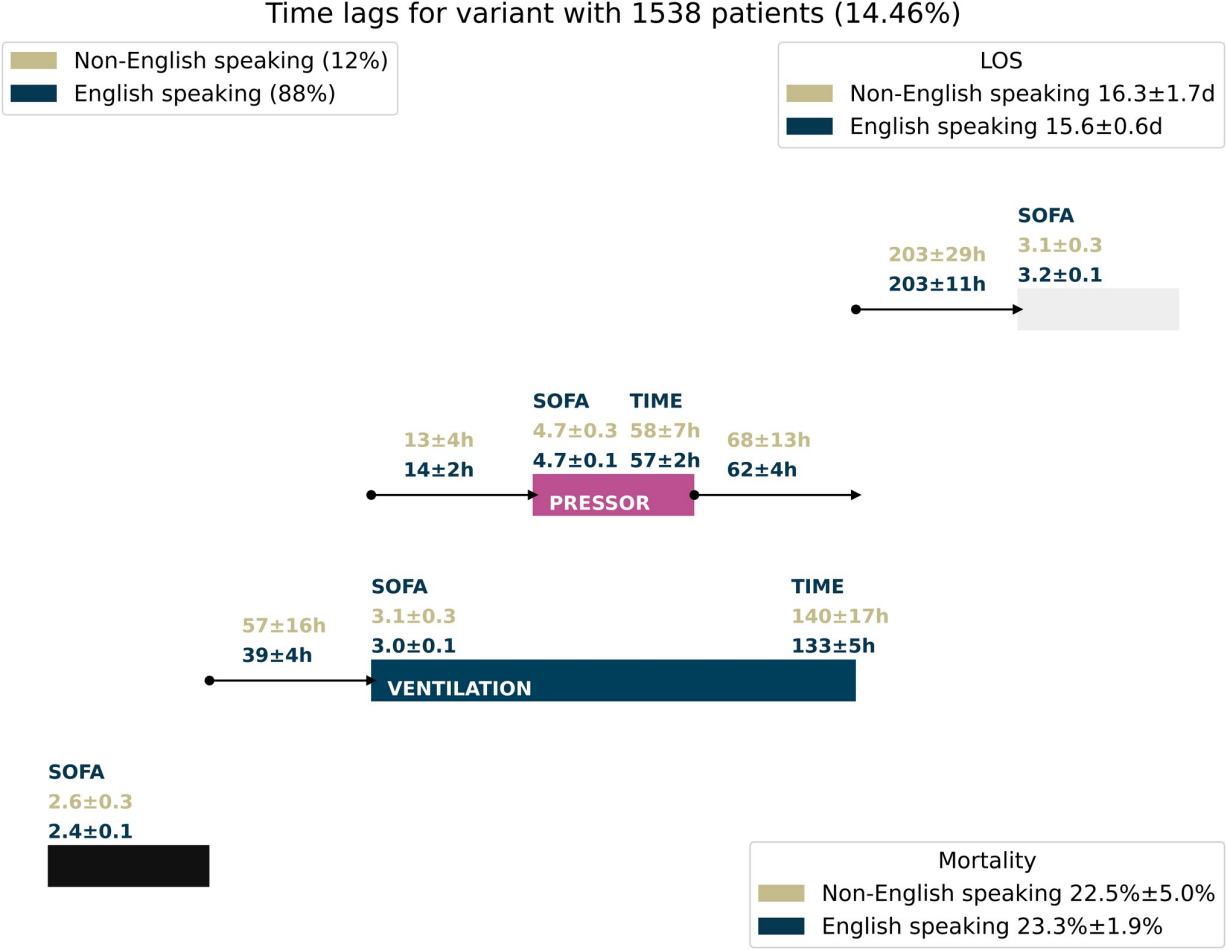
Treatment variants with metrics for English-speaking/non-English speaking patients. Average Treatment durations, time differences, and SOFA scores for one treatment variant, compared for ESP/non-ESP patients. 90% confidence intervals are depicted.

The analyzed variant with metrics split based on patient language is depicted in Fig 3. The SOFA scores at admission are in a similar range. However, the time to ventilation is higher for non-ESP patients. After that, the remaining treatment times and times between treatments all cover similar ranges, with non-ESP confidence intervals being slightly wider. While the other treatment steps and times between treatments do not show significant differences, the time to ventilation stands out as taking longer for non-ESP patients. We depict the same variant split for patient ethnicity in S16 Fig to compare for confounding effects. The differences in the time to ventilation are not associated to large disparities for the outcomes of mortality and length of hospital stay.

## Discussion

We provided two main contributions in this paper: the proposed process mining framework and its application to sepsis patients uncovering inequities. First, we discuss conditions for the general application of the framework and how our framework subsumes existing care disparities research. Second, we contextualize the findings from applying our framework to MIMIC with existing literature.

In the broader scope of health inequities, there are multiple sources of disparity that have been investigated and confirmed. While our framework can be used to systematically analyze inequities stemming from the care process, the framework cannot be used to assess other sources of inequity, e.g., structural inequities.

The most central limitation for the practical application of our framework is the data requirements. We split the patients according to their treatment variant. Naturally, this increases the data requirements compared to a traditional approach considering all patients together. To achieve a certain statistical robustness, there needs to be a minimum number of patients within a treatment variant. When controlling for confounders and comorbidities, the data must be split. This further increases the necessary sample sizes per variable. Therefore, our more granular analysis comes at the cost of larger data requirements than traditional approaches. However, the framework is designed for use with large-scale electronic health records which helps in meeting the necessary data requirements. Using the framework on a national or global scale would require standardized data integration, which would help achieve the necessary data requirements. Such standardized data integration would also help low and middle-income countries apply the analysis by avoiding the technical components and, thus, reducing required resources.

Our framework considers bias analysis on three different levels: single treatment steps, multiple treatment steps, and global treatment. Individual studies have been proposed to uncover specific biases in each of these three levels. In the following paragraphs, we discuss how these studies fit into our framework and, therefore, how our framework subsumes existing research.

The following are specific biases described in the literature and can be calculated as single-treatment step biases. First, it has been investigated whether the administration of drugs such as antibiotics adheres to guidelines [8, 12]. Such guideline adherence is checked by comparing the attributes of a single treatment step, e.g., the administered dose or the type of antibiotics, to the ones specified in the guidelines. Second, some treatment steps are associated with better outcomes if conducted by a trained specialist, which is called subspecialty care [15]. The corresponding person who conducted a treatment step would be recorded as an attribute, making it possible to assess the competency of the healthcare provider. Third, the administered amount of drugs, e.g., antibiotics, was used to explain differences in outcomes between races [24].

The most important multiple-treatment step bias that has been investigated are time differences between treatment steps, especially the time to antibiotics [8, 9, 12] or the time to resuscitation [25, 26]. This describes the time differences between two (or potentially more) treatment steps, e.g., between being admitted to the ICU and receiving the first dose of antibiotics. Another area of research is assessing subspecialty care across multiple treatment steps [15].

So far, global treatment biases with respect to the treatment process have not been explored. We present a collection of standard process mining algorithms and what biases they can uncover. By comparing the distribution of treatment variants one can uncover whether there are certain variants that are less pronounced in certain patient groups, e.g., where a treatment step is absent. This can be used to analyze the frequency of different treatment steps, such as ICU transfer, referral to treatments, or specific treatment steps [13, 27, 28], receiving preventive care [14] or emergency department admission [29] or discharge events after cardiac arrest [30]. This global view can also be used to explore patterns and global trends between patient groups’ treatment. Techniques from local process mining [31] or pattern mining [32] could point to frequent sub-treatment patterns; while techniques from process discovery could uncover differences in the global models that describe possible treatment pathways between patient groups [20]. Concept drift detection methods could investigate whether processes of different patient groups developed in the same way [33].

In our application of the framework, we found that there is a longer time to the first treatment, i.e., ventilation, for non-ESP patients while there is no significant difference for male/female patients. The times to treatments have been studied as one source of disparities [8, 9, 12]. Our findings confirm that such disparities are present in non-ESP patients. These might be explained by the language barrier which would necessitate further language capabilities in the healthcare system.

There are several limitations in our application of process mining for health disparity evaluation for sepsis care in MIMIC-IV ICU patients. First, as language is confounded with race/ethnicity we cannot exclude a racial influence on the time to ventilation. Specifically, Hispanic patients are 21% of the non-English speaking patients, compared to only 1% of English-speaking patients. Therefore, the root cause for longer times to ventilation for non-English speaking patients could also be due to a large share of patients being Hispanic or a confounding factor between both. The same holds for age. Second, since we had less data for non-ESP patients. This leads to wider confidence intervals as the variance of the estimated distribution mean is dependent on the sample sizes. Third, we grouped several similar highly influential comorbidities to control for them. Picking them out individually might increase the reliability of results, however, it also requires more data. Fourth, the reliance on electronic health records might introduce selection bias in itself, as certain important information might not be recorded within the information system. A general limitation of this study is the reliance on a single-center database. While our application on MIMIC functions as a proof of concept, future application of our framework to different databases should demonstrate the generalizability of this framework. All in all, these limitations highlight the necessity for large amounts of data to retrieve reliable results. Large studies across multiple hospitals are required to answer the questions of disparities.

Applying our framework requires the execution of several steps. First of all, we extracted the event log from the electronic health records. On the one hand, this requires knowledge of data extraction and transformation. On the other hand, this also requires medical knowledge to identify relevant treatment steps. Therefore, event log extraction necessitates an interdisciplinary collaboration between data scientists and medical staff. Later steps of the framework require further collaboration to provide a medically and statistically sound interpretation of the results.

We introduced a framework to assess biases and disparities in treatment processes using process mining in this paper. The framework builds upon two main steps: the extraction of an event log, providing sequences of treatments for patients, and treatment variant analysis, comparing the global allocation of treatment sequences as well as the differences between groups within the same treatment variant. The framework subsumes existing approaches to uncover care disparities. It provides a general resource that helps researchers answer questions about inequities in care delivery. To demonstrate this, we apply our framework to ICU patients in MIMIC-IV diagnosed with sepsis. We found that non-English-speaking patients had longer times to the first treatment, i.e., ventilation, while there were no significant sex differences in the time to first treatment. Our framework can become part of the systematic efforts to tackle health inequities and present an additional toolkit to implement monitoring measures required by the US government at the individual and global levels. Future work can integrate other aspects of process mining into a comparative framework, such as conformance checking for guideline adherence.

## Supporting information

S1 Fig Inclusion and Exclusion of MIMIC-IV patients into the study. (TIFF)

S2 Fig Distribution of treatment variants over male/female patients. (TIFF)

S3 Fig Distribution of treatment variants over English/non-English speaking patients. (TIFF)

S4 Fig Most frequent variant split according to patient sex. (TIFF)

S5 Fig Second most frequent variant split according to patient sex. (TIFF)

S6 Fig Fourth most frequent variant split according to patient sex. (TIFF)

S7 Fig Fifth most frequent variant split according to patient sex. (TIFF)

S8 Fig Additional variant containing dialysis: Seventh most frequent variant split according to patient sex. (TIFF)

S9 Fig Most frequent variant split according to patient language. (TIFF)

S10 Fig Second most frequent variant split according to patient language. (TIFF)

S11 Fig Fourth most frequent variant split according to patient language. (TIFF)

S12 Fig Fifth most frequent variant split according to patient language. (TIFF)

S13 Fig Additional variant containing dialysis: Seventh most frequent variant split according to patient language. (TIFF)

S14 Fig Most frequent variant split according to patient ethnicity. (TIFF)

S15 Fig Second most frequent variant split according to patient ethnicity. (TIFF)

S16 Fig Third most frequent variant split according to patient ethnicity. (TIFF)

S17 Fig Fourth most frequent variant split according to patient ethnicity. (TIFF)

S18 Fig Fifth most frequent variant split according to patient ethnicity. (TIFF)

S19 Fig Additional variant containing dialysis: Seventh most frequent variant split according to patient ethnicity. (TIFF)

S20 Fig Most frequent variant split according to patient insurance. (TIFF)

S21 Fig Second most frequent variant split according to patient insurance. (TIFF)

S22 Fig Third most frequent variant split according to patient insurance. (TIFF)

S23 Fig Fourth most frequent variant split according to patient insurance. (TIFF)

S24 Fig Fifth most frequent variant split according to patient insurance. (TIFF)

S25 Fig Additional variant containing dialysis: Seventh most frequent variant split according to patient insurance. (TIFF)

## References

[1] Epstein Becker Green. Biden Administration Prioritizes Health Equity in Proposed Reforms to Medicare Managed Care; Accessed on October 19, 2023. Available from: https://www.ebglaw.com/insights/biden-administration-prioritizes-health-equity-in-proposed-reforms-to-medicare-managed-care/.

[2] The Joint Commission. Joint Commission Health Equity Certification; Accessed on October 19, 2023. Available from: https://www.modernhealthcare.com/accreditation/joint-commission-health-equity-certification.

[3] Alexander GR, Kogan MD, Nabukera S. Racial differences in prenatal care use in the United States: are disparities decreasing? American Journal of Public Health. 2002;92(12):1970–1975. doi: 10.2105/ajph.92.12.1970 12453818 PMC1447361

[4] Ponce NA, Hays RD, Cunningham WE. Linguistic disparities in health care access and health status among older adults. Journal of General Internal Medicine. 2006;21(7):786–791. doi: 10.1111/j.1525-1497.2006.00491.x 16808783 PMC1924691

[5] Agency for Healthcare Research and Quality (AHRQ). National Healthcare Quality and Disparities Report, 2022; 2022. Available from: https://www.ahrq.gov/sites/default/files/wysiwyg/research/findings/nhqrdr/2022qdr.pdf.

[6] McGowan SK, Sarigiannis KA, Fox SC, Gottlieb MA, Chen E. Racial Disparities in ICU Outcomes: A Systematic Review. Critical Care Medicine. 2022;50(1):1–20. doi: 10.1097/CCM.0000000000005269 34636803

[7] Frei CR, Mortensen EM, Copeland LA, Attridge RT, Pugh MJ, Restrepo MI, et al. Disparities of care for African-Americans and Caucasians with community-acquired pneumonia: a retrospective cohort study. BMC Health Services Research. 2010;10:143. doi: 10.1186/1472-6963-10-143 20507628 PMC2890642

[8] Mayr FB, Yende S, D’Angelo G, Barnato AE, Kellum JA, Weissfeld L, et al. Do hospitals provide lower quality of care to black patients for pneumonia? Critical Care Medicine. 2010;38(3):759–765. doi: 10.1097/CCM.0b013e3181c8fd58 20009756 PMC3774066

[9] Madsen TE, Napoli AM. Analysis of Race and Time to Antibiotics Among Patients with Severe Sepsis or Septic Shock. Journal of Racial and Ethnic Health Disparities. 2017;4(4):680–686. doi: 10.1007/s40615-016-0271-7 27553054

[10] Agerström J, Carlsson M, Bremer A, Herlitz J, Rawshani A, Årestedt K, et al. Treatment and survival following in-hospital cardiac arrest: does patient ethnicity matter? European Journal of Cardiovascular Nursing. 2022;21(4):341–347. doi: 10.1093/eurjcn/zvab079 34524428

[11] Israelsson J, Carlsson M, Agerström J. A more conservative test of sex differences in the treatment and outcome of in-hospital cardiac arrest. Heart & Lung. 2023;58:191–197. doi: 10.1016/j.hrtlng.2022.12.008 36571977

[12] Mortensen EM, Cornell J, Whittle J. Racial variations in processes of care for patients with community-acquired pneumonia. BMC Health Services Research. 2004;4(1):20. doi: 10.1186/1472-6963-4-20 15304197 PMC514714

[13] Tyler PD, Stone DJ, Geisler BP, McLennan S, Celi LA, Rush B. Racial and Geographic Disparities in Interhospital ICU Transfers. Critical Care Medicine. 2018;46(1):e76–e80. doi: 10.1097/CCM.0000000000002776 29068859 PMC5743219

[14] Siegel R, Naishadham D, Jemal A. Cancer statistics, 2013. CA: A Cancer Journal for Clinicians. 2013;63(1):11–30. 23335087 10.3322/caac.21166

[15] Breathett K, Liu WG, Allen LA, Daugherty SL, Blair IV, Jones J, et al. African Americans Are Less Likely to Receive Care by a Cardiologist During an Intensive Care Unit Admission for Heart Failure. JACC: Heart Failure. 2018;6(5):413–420. doi: 10.1016/j.jchf.2018.02.015 29724363 PMC5940011

[16] van der Aalst WMP. Process Mining—Data Science in Action, Second Edition. Springer; 2016. Available from: 10.1007/978-3-662-49851-4.

[17] Helbig K, Römer M, Mellouli T. A Clinical Pathway Mining Approach to Enable Scheduling of Hospital Relocations and Treatment Services. In: Motahari-Nezhad HR, Recker J, Weidlich M, editors. Business Process Management—13th International Conference, BPM 2015, Innsbruck, Austria, August 31—September 3, 2015, Proceedings. vol. 9253 of Lecture Notes in Computer Science. Springer; 2015. p. 242–250. Available from: 10.1007/978-3-319-23063-4_17.

[18] Gerhardt R, Valiati JF, dos Santos JVC. An Investigation to Identify Factors that Lead to Delay in Healthcare Reimbursement Process: A Brazilian case. Big Data Res. 2018;13:11–20. doi: 10.1016/j.bdr.2018.02.006

[19] Chiudinelli L, Dagliati A, Tibollo V, Albasini S, Geifman N, Peek N, et al. Mining post-surgical care processes in breast cancer patients. Artif Intell Medicine. 2020;105:101855. doi: 10.1016/j.artmed.2020.101855 32505422

[20] Caron F, Vanthienen J, Vanhaecht K, van Limbergen E, Weerdt JD, Baesens B. Monitoring care processes in the gynecologic oncology department. Comput Biol Medicine. 2014;44:88–96. doi: 10.1016/j.compbiomed.2013.10.015 24377692

[21] Cho M, Kim K, Lim J, Baek H, Kim S, Hwang H, et al. Developing data-driven clinical pathways using electronic health records: The cases of total laparoscopic hysterectomy and rotator cuff tears. Int J Medical Informatics. 2020;133. doi: 10.1016/j.ijmedinf.2019.104015 31683142

[22] Kim HY. Statistical notes for clinical researchers: Chi-squared test and Fisher’s exact test. Restorative Dentistry & Endodontics. 2017;42(2):152–155. doi: 10.5395/rde.2017.42.2.152 28503482 PMC5426219

[23] Johnson A, Bulgarelli L, Pollard T, Horng S, Celi LA, Mark R. MIMIC-IV; 2023. PhysioNet. Available from: 10.13026/6mm1-ek67.

[24] Merchant RM, Becker LB, Yang F, Groeneveld PW. Hospital racial composition: a neglected factor in cardiac arrest survival disparities. American Heart Journal. 2011;161(4):705–711. doi: 10.1016/j.ahj.2011.01.011 21473969 PMC3073775

[25] Agerström J, Carlsson M, Bremer A, Herlitz J, Israelsson J, Årestedt K. Discriminatory cardiac arrest care? Patients with low socioeconomic status receive delayed cardiopulmonary resuscitation and are less likely to survive an in-hospital cardiac arrest. European Heart Journal. 2021;42(8):861–869. doi: 10.1093/eurheartj/ehaa954 33345270 PMC7897462

[26] Al-Dury N, Rawshani A, Karlsson T, Herlitz J, Ravn-Fischer A. The influence of age and gender on delay to treatment and its association with survival after out of hospital cardiac arrest. The American Journal of Emergency Medicine. 2021;42:198–202. doi: 10.1016/j.ajem.2020.11.033 33234358

[27] Mehta LS, Beckie TM, DeVon HA, Grines CL, Krumholz HM, Johnson MN, et al. Acute Myocardial Infarction in Women: A Scientific Statement From the American Heart Association. Circulation. 2016;133(9):916–947. doi: 10.1161/CIR.0000000000000351 26811316

[28] Winther-Jensen M, Hassager C, Kjaergaard J, Bro-Jeppesen J, Thomsen JH, Lippert FK, et al. Women have a worse prognosis and undergo fewer coronary angiographies after out-of-hospital cardiac arrest than men. European Heart Journal Acute Cardiovascular Care. 2018;7(5):414–422. doi: 10.1177/2048872617696368 29064270

[29] Dawson LP, Andrew E, Nehme Z, Bloom J, Biswas S, Cox S, et al. Association of Socioeconomic Status With Outcomes and Care Quality in Patients Presenting With Undifferentiated Chest Pain in the Setting of Universal Health Care Coverage. Journal of the American Heart Association. 2022;11(7):e024923. doi: 10.1161/JAHA.121.024923 35322681 PMC9075482

[30] Chan PS, Nichol G, Krumholz HM, Spertus JA, Jones PG, Peterson ED, et al. Racial differences in survival after in-hospital cardiac arrest. JAMA. 2009;302(11):1195–1201. doi: 10.1001/jama.2009.1340 19755698 PMC2795316

[31] Tax N, Sidorova N, Haakma R, van der Aalst WMP. Mining local process models. J Innov Digit Ecosyst. 2016;3(2):183–196. doi: 10.1016/j.jides.2016.11.001

[32] Chapela-Campa D, Mucientes M, Lama M. Mining frequent patterns in process models. Inf Sci. 2019;472:235–257. doi: 10.1016/j.ins.2018.09.011

[33] Adams JN, Pitsch C, Brockhoff T, van der Aalst WMP. An Experimental Evaluation of Process Concept Drift Detection. Proc VLDB Endow. 2023;16(8):1856–1869. doi: 10.14778/3594512.3594517

