# Supplementary figures and images for: A health equity monitoring framework based on process mining
Source: PLOS Digit Health. 2024 Aug 28;3(8):e0000575. doi: 10.1371/journal.pdig.0000575 (PMC11355534; doi:10.1371/journal.pdig.0000575)

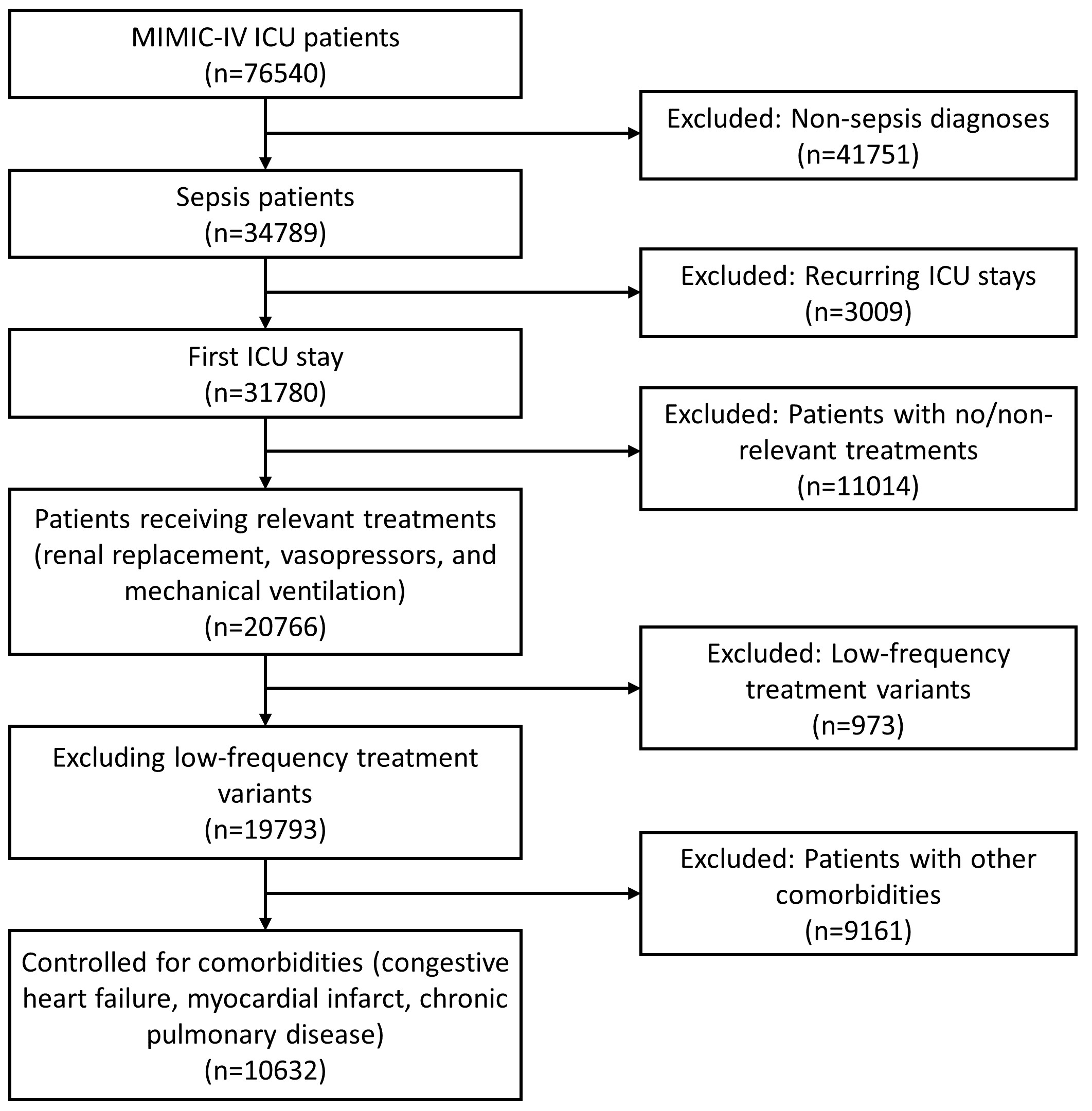

Supplement: S1 Fig — (TIFF) [file pdig.0000575.s001.tiff]

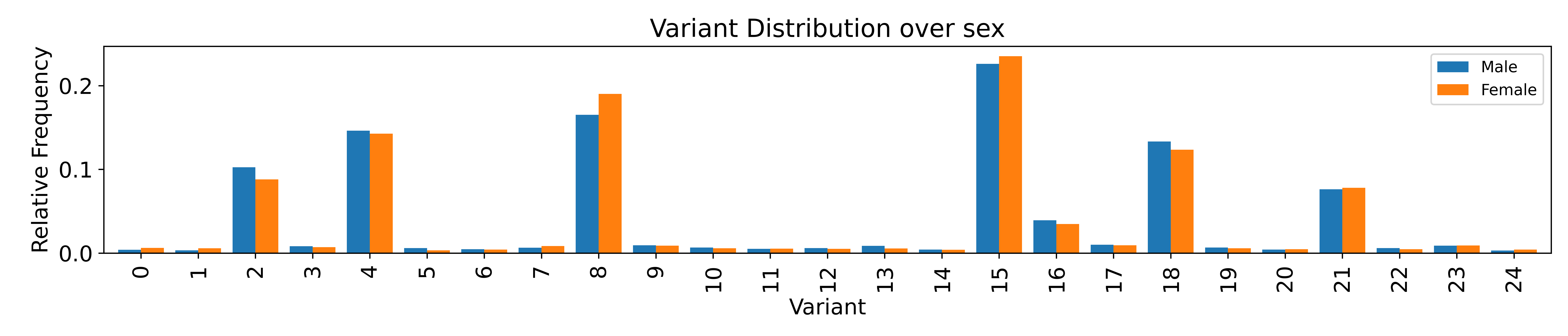

Supplement: S2 Fig — (TIFF) [file pdig.0000575.s002.tiff]

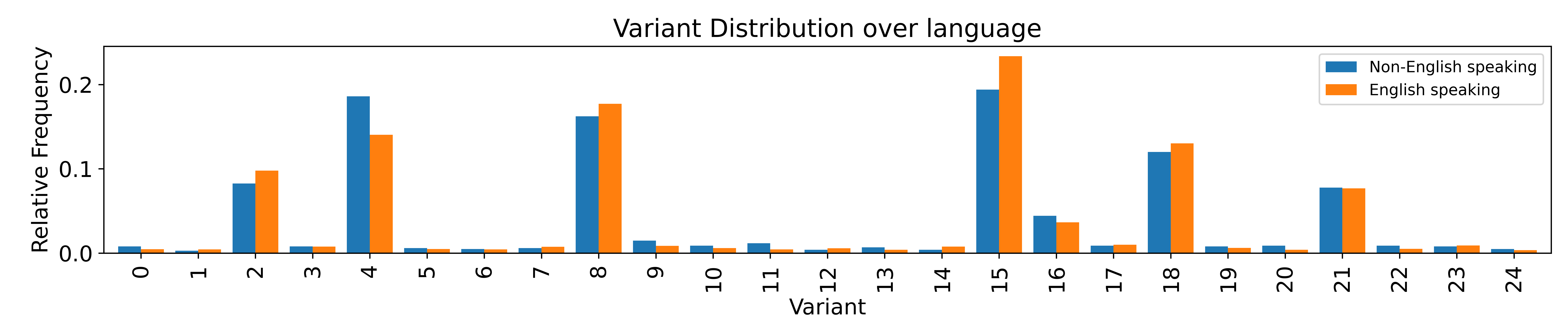

Supplement: S3 Fig — (TIFF) [file pdig.0000575.s003.tiff]
